# To Steal or Not to Steal: Self-Discrepancies as a Way to Promote Pro-social Behavior: The Moderating Role of Self-Interest

**DOI:** 10.3389/fpsyg.2022.748298

**Published:** 2022-04-27

**Authors:** Alin Gavreliuc, Dana Gavreliuc, Alin Semenescu

**Affiliations:** ^1^Department of Psychology, West University of Timișoara, Timișoara, Romania; ^2^Teacher Training Department, West University of Timișoara, Timișoara, Romania; ^3^Institute for Advanced Environmental Research (ICAM), West University of Timișoara, Timișoara, Romania

**Keywords:** pro-social behavior, stealing, field experiment, post-communism, Romania

## Abstract

Previous research showed that acting immorally on one occasion can determine a greater availability for pro-social behavior on a subsequent occasion. Nevertheless, moderating factors for this effect, such as financial interest remained largely unexplored. The present field experiment (*N* = 587) was organized in an urban setting, in a post-communist society (Romania), in a context of public anonymity and examined passersby’s pro-social behavior on two consecutive occasions. The procedure involved a confederate “losing” a banknote of different values (1, 10, 50, 100, or 500 RON), which invited passersby’s pro-social behavior to return it (or not). Participants who decided to steal the banknote were approached by a second confederate and asked politely to return the banknote. Our research was articulated mainly as a quantitative approach by measuring participants’ pro-social behavior toward the person who lost the banknote, their subsequent pro-social behavior toward the confederate who exposed their behavior and the number of words they produced during a post-experimental interview in which they could justify their behavior. At the same time, we also performed a qualitative approach, through which we explored the themes evoked in their justifications and their relation with their previous behavior. Results indicate a moderating effect of economic interest on pro-social behavior toward the confederate who lost the banknote, as well as on their subsequent pro-social behavior toward the second confederate. Participants who stole the banknote also used significantly more words to justify their behavior, and this tendency could be observed especially in the case for higher values of the banknote. Results are critically discussed in a context dominated by an inherited pattern of distrust and social cynicism.

## Introduction

Pro-social behavior has been considered an essential contributor to social welfare (Piliavin et al., 1981; Batson and Powell, 2003; Weinstein and Ryan, 2010; Wittek and Bekkers, 2015; Smith, 2019). Its role in generating interpersonal and societal wellbeing has been shown at the individual (Henrich et al., 2001; Weinstein and Ryan, 2010), group (Busching and Krahé, 2020), and societal levels (Levine et al., 2001; Knafo et al., 2009; Smith, 2019). The scientific literature focused on pro-social behavior describes it as an interpersonal act between a benefactor and a receiver of the action, which a particular society defines as beneficial to other people or the social order (Dovidio et al., 2017, p. 17). Thus, pro-social behavior is like the “social glue,” which emerges in interpersonal interactions and encourages living together peacefully and productively (Lay and Hoppmann, 2015).

The literature distinguishes between altruistic and egoistic motives of pro-social behavior (Frazier et al., 2013; Feigin et al., 2014). Egoistic motives are centered around the own interest of the person involved in the social interaction, such as the need for self-esteem and a positive self-image, for increasing his/her social status, or for managing negative emotions associated with the situation (e.g., anxiety, fear, sadness, or guilt). On the other hand, altruistic motives are generated by a genuine desire to support others without seeking any benefits for oneself (Penner et al., 2005). Thus, altruism is an entirely other-oriented and generous way of thinking and acting that proves to be beneficial both for the recipients and for society (Snyder and Dwyer, 2013).

One way of studying pro-social behavior is by involving individuals in situations in which they have the opportunity to act honestly or to cheat. The literature on this topic is extensive, yet studies that go beyond the strict confines of the laboratory space and investigate real-life situations, while still maintaining a high methodological quality, are surprisingly rare (see Gomes et al., 2021). Moreover, most of the experimental literature is based on procedures in which participants are aware that they are being observed, with low financial stakes involved and conducted mostly on Western, educated, rich and democratic populations (Cohn et al., 2019). Consequently, in the present study, we addressed these shortcomings by conducting a field experiment, in which we created a scenario of interaction with unaware participants, through which we tested pro-social behaviors in the context of everyday life context with ostensibly high financial stakes, in a highly underrepresented culture (Romania). Our scenario is similar to others applied in cross-cultural studies (e.g., Falk et al., 2018) and involves a situation in which participants unexpectedly find some money on the street and have to decide whether they act pro-socially and return it to the person who lost it or appropriate it. This decision is soon followed by another situation in which participants who initially stole the money are made aware of their unethical behavior and have to decide again whether they return or keep the money for themselves. Unlike other approaches however, the present study used both a quantitative as well as a qualitative methodology, to better understand the particularity of pro-social behaviors when a spontaneous need for help is activated in the public space.

## Literature and Theoretical Background

Several factors have been shown to be associated with pro-social behavior, among which the characteristics of the help-provider (e.g., Wollman et al., 1980; Gire and Williams, 2007), the characteristics of the help-seeker (e.g., Milgram et al., 1965; Simmons and Zumpf, 1983), situational factors (e.g., Newman, 1979; Keizer et al., 2008), or cultural variables (e.g., Vives and FeldmanHall, 2018; Cohn et al., 2019). Concerning the impact of situational factors, which is the main focus of the present study, one relevant theoretical framework that was used to explain differences in pro-social behavior when financial stakes are involved is the *subjective expected utility theory* (SEUT; Savage, 1954, Farrington, 1979, Farrington et al., 2020). SEUT describes how in a risk situation (like a specific context in which a person is confronted with a potential gain that can be obtained dishonestly), the person involved activates a behavioral decision based on: (a) utility (the subjective benefit or attractiveness of the potential gain), (b) subjective costs (the threat or sense of apprehension of being “discovered”), and (c) the probabilities related to them. By pondering all these factors, the decision-maker selects the behavioral alternative with the highest subjective expected utility and acts accordingly.

The results of several empirical studies support the validity of this theoretical framework. For example, a classical study conducted by Merritt and Fowler (1948) tested whether letters containing visible money were returned or not by pedestrians from East and Midwest cities in the US, by comparing the rate of return with that of “normal” letters containing only a simple visible message on a sheet of paper. Results showed that 85% of the letters were returned in the “normal” condition and only 54% in the money condition. More than that, in the money condition, 11 out of 54 letters were returned opened. Thus, when ordinary people are confronted with an invitation to help an unknown person, the majority of Americans acted pro-socially, yet only about half did so when their own immediate gain was also involved. Gabor and Barker (1989) also used the “lost letter” technique in Canada and observed that almost a quarter of all participants failed to return an envelope containing $150 (measured as stealing behavior).

Using a similar paradigm, Penner et al. (1976) tested the dispute between situational and personal factors in predicting reactions to “lost” money (returning, ignoring or taking) by an identifiable person (because of his wallet), by an unknown person belonging to a certain group (a person from the psychology department) or by an unidentifiable owner, in three different contexts: a psychology laboratory, a testing room from campus where evaluation services were provided and an impersonal place, like a campus washroom. Results showed that people’s decision to return the money was influenced by the characteristics of the person who lost the money and by the context, whereas personality had almost no influence on behavior. Based on a cost analysis associated with the bystander effect (Piliavin et al., 1975), the authors argued that the harder it was to identify the real owner of the money and the more impersonal the contexts were (and thus the lower the probability of being sanctioned was), the less pro-social was participants’ behavior. Similarly, Newman (1979) tested the role of familiarity with the context and the value of money and observed their influence on the rate of returning “lost” money. Results showed that non-familiar and impersonal places (like a central shopping area) were more likely to induce stealing and that in these places stealing increased proportionally with the value of money. In another field experiment, Keizer et al. (2008) showed that cues of norm violation (i.e., garbage bags in the vicinity of a mailbox) impacted passersby’s pro-social behavior. They evidenced an increase of stealing an envelope visibly containing money in “disordered” settings, in which other norms were previously violated and thus in which the perceived probability of being caught was lower.

Despite the reviewed examples, field experiments for testing pro-social behavior are relatively rare, even though studying people’s pro-social behavior in everyday life is frequently suggested as a way to go beyond the controlled environment of laboratories (Lay and Hoppmann, 2015). For instance, Gomes et al. (2021) conducted a systematic review on stealing and monetary dishonesty incorporating 40 years of research (between 1979 and 2019) and found only 14 field experiments conducted in the area of Psychology/Social Sciences, of which only one was carried out also in ex-communist countries (i.e., Cohn et al., 2019). Even though the majority of these studies showed that higher levels of financial benefits and lower probabilities of being caught anticipated lower levels of pro-social behavior, there was also an opposite tendency that appeared in some studies, in which higher potential benefits determined higher levels of pro-social behavior in some circumstances (see also Mazar et al., 2008). For example, Cohn et al. (2019) tested the influence of self-interest on pro-social behavior in 355 different cities across 40 national cultures, in large field experiments involving more than 17,000 participants. The procedure involved a “lost” wallet in different public places (e.g., museums and post offices), containing a business card, in two conditions: with 13.45$ inside (money condition) or without money inside (no money condition), which was “found” by a confederate. The confederate then asked an employee of these public spaces to return it, because he/she was in a hurry, and left it on the counter. In contrast to the self-interest evidenced in most studies, in 38 out of the 40 national cultures the presence of money inside the wallet increased the rate of return. Furthermore, in another study organized in the United Kingdom, United States, and Poland, the authors manipulated the sum of money inside the wallet (introducing a “big money” condition—94.14$) and observed a further increase of this tendency, with the rate of return being the highest in the “big money” condition. A similar result was obtained by Azar et al. (2013), who found that customers of a restaurant were more likely to return a higher amount of excessive change (about 12$) than a smaller amount (about 3$). The inconsistency between such results and those initially reviewed suggests an interaction between costs and benefits that needs to be investigated further (Gomes et al., 2021). Thus, one of the aims of the present study is to contribute to the body of existing literature by investigating, through a field experiment, the influence of the costs–benefits mechanism on pro-social behavior, in anonymity conditions, when high financial stakes are involved and in a new cultural context (post-communist Romania).

### Self-Discrepancies and Pro-social Behavior

While previous studies investigated the influence of self-interest, another direction of research focused on the role of moral inconsistencies in pro-social behavior. Moral consistency is defined by Campbell (2017) as “responding morally in a similar way to cases that are morally alike.” Consequently, if one decides to follow one’s own interest in a particular situation, to be morally consistent, then one should do the same in all similar situations. Nevertheless, research shows that this is frequently not the case. For instance, Otto and Bolle (2020) found in their experiment that, for those participants that decided to engage in stealing, half continued to follow their own interest while the other half engaged in pro-social behavior in their next immediate similar decision. Furthermore, several studies found that immoral behavior in one situation can even encourage a higher degree of moral behavior on a subsequent occasion (e.g., Jordan et al., 2011; Mulder and Aquino, 2013) as an act of moral cleansing (Zhong and Liljenquist, 2006) and this may be because of the negative feelings, such as shame, guilt, anger, or a threat to self-image that one may experience (Mazar et al., 2008; Bonner et al., 2017). A relevant theoretical framework that can account for this relationship is *self-discrepancy theory* (Higgins, 1987; see also Barnett et al., 2017) which, like other “inconsistency” theories, such as cognitive dissonance (Festinger, 1957), self-consistency (Lecky, 1945), or incongruity (Osgood and Tannenbaum, 1955), proposes that people are motivated to avoid inconsistencies. The theory distinguishes between one’s *self-concept* (how the self is currently represented) and one’s *self-guides*, which are standards that are yet to be achieved. Self-discrepancy theory postulates that people are motivated to find themselves in a condition in which their self-concept is congruent with their self-guides, because discrepancies generate discomfort.

In the moral domain, self-discrepancy theory posits that people maintain a state of equilibrium by behaving in ways that adhere to internalized moral standards or the standards of important others (Higgins, 1997). Therefore, instances of immoral behavior that violate such standards should generate discomforting thoughts and emotions, as a result of discrepancies between the actual self-concept and self-guides. However, discomfort is felt only when discrepancies are made accessible (Higgins, 1987; see also Duval and Wicklund, 1972), which highlights the role of situational factors as particularly important. For instance, Higgins et al. (1986) showed that, for people who were highly discrepant, priming their discrepancies lead to the experience of negative emotions, such as dejection and agitation. Therefore, activating discrepancies in people who acted immorally on one occasion can trigger negative emotions that can motivate them to restore congruence by behaving morally on a subsequent occasion. For example, in a classic study, Carlsmith and Gross (1969) showed that participants who delivered painful electric shocks to a confederate were more likely to comply with a pro-social request to help prevent the construction of a freeway through redwood trees in Northern California than participants in the control condition. Similarly, Sachdeva et al. (2009) showed that people who activated an image of themselves as immoral persons donated five times more money to charity than those who activated the image of a moral person. Jordan et al. (2011) evidenced an increase in pro-social intentions and a decrease in cheating behavior for those individuals who recalled an instance of immoral behavior, compared to those who recalled moral or neutral events, while Dai et al. (2018) showed that people who had just paid a fine for riding the public transport without a ticket acted more honestly than other fare-dodgers who were not caught.

Even though this effect is well-researched, it goes without saying that not everyone will have the same degree of motivation to reduce discrepancies. Not all people who behave immorally will have the drive to restore moral congruence and not all that do will actually engage in compensatory behaviors on all occasions. Thus, the question that arises is for whom and in what circumstances this effect takes place? While individual differences, such as moral identity, have been shown to moderate the relationship between immoral acts and subsequent moral behavior by determining a stronger compensatory reaction (Mulder and Aquino, 2013; Ding et al., 2016), the role of situational factors in this relationship, such as self-interest, in not yet clear. Based on SEUT, it is likely that in low-gain conditions people may prioritize congruence restoration after a moral transgression, while they may be more ready to incur the cost of self-discrepancies when their reward for persisting in dishonesty is higher. Therefore, a second goal of the present study is to investigate self-interest (in the form of financial gain) as a moderator of engagement in subsequent moral behavior after a moral discrepancy is activated.

Further, when self-discrepancies are publicly revealed, people may also be motivated to engage in a process of self-image negotiation called *facework* (Ting-Toomey, 1988; Zhang et al., 2014), which refers to the communicative strategies that one uses to maintain her/his positive image in social interactions (Ting-Toomey, 1988; Oetzel et al., 2001). For this purpose, they may try to explain, rationalize or excuse their behavior or may engage in deceptive strategies that allow them to maintain a positive “face” in the eyes of others. Whatever the strategy, we argue that engaging in such facework is more socially and cognitively demanding compared with the situation when no self-justification is needed (i.e., no self-discrepancy is activated) and that this extra effort is reflected verbally in the volume of explanations that people produce when they are required to offer an explanation for their inconsistencies. Thus, a third goal of the present study is to investigate how self-discrepancies affect people’s verbal behavior.

### The Present Study

The present study aims to present new evidence on the moderating role of self-interest on pro-social behavior in an everyday life context, to explore how it influences individual behavior after self-discrepancies are activated and to find out how people respond verbally when inconsistencies in their behavior are made salient. For this purpose, we used both a quantitative approach through which we manipulated the potential gain of the participants and measured their concrete pro-social behavior on two consecutive occasions and the number of words they used to justify it, as well as a qualitative analysis of participants’ interviews, in which they explained their behavior. More concretely, we created a scenario in which we investigated passersby’s pro-social behavior by arranging that a banknote of different values is “lost” in front of them, in a public space, and monitored their behavior. For those who initially stole the banknote, self-discrepancy was activated by a confederate who revealed their behavior and offered them a second chance to return the banknote. Soon after, participants were requested to explain their behavior, in their own words, in a short interview. Based on SEUT, self-discrepancy theory and previously reviewed studies, we expect that:

*Hypothesis* 1: Participants’ pro-social behavior toward the person losing the banknote decreases when their potential gain increases.

*Hypothesis* 2: For those participants who decide to steal the banknote, subsequent pro-social behavior decreases when their potential gain increases. In other words, participants’ self-interest will moderate the relationship between self-discrepancies and subsequent moral behavior.

*Hypothesis* 3: The volume of explanations provided by self-discrepant participants (i.e., those who steal the banknote) will be higher than for those who return it.

In an explorative manner, we will also analyze participants’ verbal explanations and how these relate to their previous behavior (i.e., returning or stealing the banknote).

## Materials and Methods

### Procedure

The present study (“the fast-handed passerby”) involved an interaction between a confederate, an aged man (around 65 years old) appearing to come from a poor background and naïve pedestrians. The place of interaction was in a supermarket’s vicinity, in the city of Timisoara (approximately 350,000 inhabitants), on a relatively crowded street, placed at least 50 m from the entrance of the supermarket. In each new trial, when a pedestrian spontaneously passed by him, the confederate passed his coat over his shoulder and “unexpectedly lost,” by “mistake,” a 1, 10, 50, 100, or 500 RON banknote (Romanian currency, 1 RON ≈ 0.20 euros, photocopied from https://www.allnumis.ro/catalog-bancnote/romania, with a short mention added on it: “*This is a photocopied paper, used only for the experimental purpose in a Social Psychology field experiment study*.” The photocopied banknotes were identical to real ones and could not be recognized as fake at first sight, as evidenced in our pilot study—see an example in Supplementary Material). Participants’ pro-social behavior was monitored by a collaborator placed relatively close (around 5 m) to this spontaneous interaction, who appeared to be checking his mobile phone. It was agreed with the experimenter that the collaborator who monitored the interaction should count participants’ behavior only if: (a) the pedestrian non-ambiguously observed the whole incident and (b) she/he raised the banknote from the ground. We were interested only in the participants who did this explicit gesture and picked up the banknote, because in this way they became the holders of a resource that could be returned to the real owner, or appropriated. Moreover, their way of acting could more adequately and non-ambiguously measure pro-social behavior, without the need to speculate on their reasons if they did not intervene at all (see Lin et al., 2016). If the pedestrian just watched the incident and continued, after around 5 s, our collaborator returned the banknote to the “old man” and the new trial was prepared. Whatever the pedestrian’s behavior (to return or steal the money), after around 10 s, a second confederate politely stopped the pedestrian and explained the whole scenario, the stake of the research and asked for participant’s verbal consent. He/she also politely requested the participant to explain in a post-experimental interview (fixed at a maximum of 120 s) her/his previous behavior and to return the money, if the participant stole the banknote. At this step, two other dependent variables were measured, namely, *pro-social behavior toward the second confederate* and *face-saving behavior*.

The experiment took place during weekdays business afternoon hours, in similar locations (described before) and only in stable weather contexts, avoiding any unpleasant atmospheric conditions (like rainy or windy moments), in the same season (in spring, between March and May 2019). After each interaction, a new trial could start only after at least 10 min from the previous one, in a similar area, but not closer than 200 m from the previous place of interaction. Thus, we did not organize more than 15 trials on each day during the data collecting process.

### Variables

The independent variable (IV) was the *value of the lost banknote* (five conditions: 1, 10, 50, 100, and 500 RON). The dependent variables were: *pro-social behavior toward the first confederate* (DV1), operationalized by measuring the return rate of the banknote to the person who lost it, *pro-social behavior toward the second confederate* (DV2), operationalized by measuring the return rate of the banknote to the confederate who requested participants to return the money and *face-saving behavior* (DV3), operationalized by measuring the number of words produced by the participants in the post-experimental interviews.

### Selection Procedure

To ensure a roughly random selection, in each new trial, the 10th pedestrian was selected. If the 10th pedestrian did not fit the selection criteria, the confederate was instructed to select the next appropriate person. Criteria for selection were based on exclusion: participants were excluded if they were in a hurry, expressed any explicit distress, were accompanied by someone else (i.e., were not alone) or were involved in another task (like reading or talking on their mobile phone, etc.). Thus, each pedestrian who was not characterized by these features could become a potential participant in our study. We did not precisely count the number of participants who were rejected using these exclusion criteria, but the approximate number of them was around 1/3 of the pedestrians integrated in this field experiment.

### Calibration of the Sample

For an adequate calibration of our sample size, we performed a power analysis (PA), using G*Power, version 3.1.9.7 (Faul et al., 2007). We calculated the required sample size in order to detect small effects. Because our analyses implied chi-square tests and one-way ANOVA, we performed PA for both. Thus, for a small effect of Cohen’s *w* = 0.15 for a chi-square test with *df* = 4, *α* = 0.05 and a power of 0.80, the required sample size was *N* = 531. The PA analysis for the ANOVA test to detect a small effect size *f* = 0.15, with five groups, *α* = 0.05 and a desired power of 0.80, revealed a required sample size of *N* = 540. Based on this rationale, our global sample was established at N > 540. In our concrete design, the sample size was *N* = 587.

### Approaching the Qualitative Data

Methods, such as oral history or non-structured interviews, are useful in “giving a voice” and “making sense” of the genuine communication of participants (Larkin et al., 2006). We performed a thematic analysis on participants’ interviews, using the Interpretative Phenomenological Analysis Method (IPA; Smith and Shinebourne, 2012). IPA is suitable for integrating an insider’s perspective in explaining participants’ meanings associated with their behavior. Because it is a phenomenological interview type (Goffman, 2017), IPA can significantly enrich the understanding of the meanings generated during the interaction between the confederate and individual participants, by producing a coherent narrative as close as possible to the participant’s view (Larkin et al., 2006). After collecting all interviews, we followed the methodological recommendations for interpreting such data (see Smith et al., 2009). Thus, in the first step, we randomly selected around 15% of the interviews (*N* = 93), and a group of two experts trained in the IPA analyzed all the emerged themes and the associated subthemes. After that, the experts confronted the themes and finally agreed by consensus on seven of them, each focused on a specific semantic area. IPA is less preoccupied with the quantitative accuracy of measuring all the categories included in an interpersonal discourse, like a classical content analysis (Vaismoradi et al., 2013), and focuses more on the thematic salience of the major categories that guide the argumentative speech. In the last stage of the qualitative analysis, all interviews were analyzed (*N* = 587) based on the emerged themes. The major themes were the following: implicitly normative, explicitly/ostentatiously normative, interpersonal functional cynicism, absurd/incoherent explanations, mercy/support, recognition and assuming the mistake and non-informative message. Each participant was assigned to one of these themes, while disagreements were resolved through consensus.

### Ethics and Pilot Study

The present research was ethically approved by the Scientific Committee of the Center for Social Diagnosis from the Faculty of Sociology and Psychology of the West University of Timisoara. In requesting approval, the title, procedure, ethical implications for human participants, methods and expected results were described. Even if the pedestrians were not aware of their initial participation in the experiment, their privacy was respected during and after the experimental scenario. All naïve individuals who accepted the interaction with the confederate were debriefed at the end and asked for their consent. Before starting the actual experiment, we tested in a pilot study (*N* = 12) whether similar participants (naïve pedestrians) are likely to be distressed by the proposed scenario. None of them reported any explicit distress once they discovered the true nature of the research at the debriefing step. Also, none of the participants involved in the actual field experiment reported any explicit distress caused by their participation in the experiment. Through the pilot study we also tested the realism of the proposed scenario: Of the 12 participants involved in the pilot study, none could tell that the money used was fake.

### Statistical Analyses

Because pro-social behavior and value of the lost banknote were measured as discrete variables, we use chi-square tests to check their association, hypothesized in H1 and H2. To test H3, an independent samples t-test is conducted, to check the difference in the volume of words produced in the post-experimental interviews, between the participants who stole the banknote and those who returned it.

## Results

SPSS v.21.0 was used to conduct all analyses. Of all passersby involved in the experiment, 65.29% (587 from the total of 899) saw the lost banknote and reached down for it. To test our first hypothesis, we first performed a chi-square test to verify whether pro-social behavior toward the first confederate depended on the value of the lost banknote. Test results evidenced significant differences in pro-social behavior, depending on the value of the banknote, *χ*^2^(4) = 24.848, *p* < 0.001 (see Table 1). The influence of value on pro-social behavior toward the first confederate had an effect *V* = 0.206 which, according to Cohen (1988, p. 222) guidelines, represents a large effect.

**Table 1:** Cross-tabulation of values of the “lost” banknote and pro-social behavior toward the first confederate (*N* = 587).

Pro-social behavior (DV1)	Value of banknote						*χ* ^2^	*df*	*V*
	1 RON	10 RON	50 RON	100 RON	500 RON	Total			
Returned the banknote	87 (74.4%)	85 (69.1%)	77 (65.8%)	68 (57.6%)	51 (45.4%)	368 (62.7%)	24.848^***^	4	0.206
Appropriated the banknote	30 (25.6%)	38 (30.9%)	40 (34.2%)	50 (42.4%)	61 (54.5%)	219 (37.3%)			

*V = effect size (Cramer’s V coefficient) and RON, Romanian currency (Leu)*.

*** *p < 0.001*.

The highest rate of return was for the lowest value banknote (74.4% for 1 RON), while the lowest rate of return was for the highest value banknote (45.4% for 500 RON). The rate of pro-social behavior in the 1 RON condition was significantly higher than in the 100 RON (*Z* = 2.717, *p* = 0.003) and 500 RON (*Z* = 4.482, *p* < 0.001) conditions, but not significantly higher compared to 10 and 50 RON conditions; the rate of pro-social behavior in the 10 RON condition was significantly higher than in the 100 RON (*Z* = 1.853, *p* = 0.032) and 500 RON (*Z* = 3.674, *p* < 0.001) conditions, but not significantly higher than in the 50 RON condition; the rate of pro-social behavior in the 50 RON condition was significantly higher than in the 500 RON (*Z* = 3.107, *p* < 0.001) condition, but not significantly higher than in the 100 RON condition, while that in the 100 RON condition was significantly higher than in the 500 RON (*Z* = 1.851, *p* = 0.032) condition. Thus, when the potential gain is small (1, 10, and 50 RON conditions), the decrease in pro-social behavior is rather small and non-significant, but as soon as it becomes substantial (100 or 500 RON), results illustrate a progressive decrease in pro-social behavior. Therefore, the data supports our first hypothesis (H1).

To test our second hypothesis, we conducted the analysis only on the subsample of participants who initially stole the banknote (*N* = 219). In the sequence called “the moment of truth,” when participants were approached by the second confederate and their previous behavior was revealed, participants could decide either to return the stolen money or to definitively appropriate them. When self-discrepancies were activated, 198 of the 219 participants (90.4%) that initially stole the banknote, decided to return it. However, this rate was not equally distributed across conditions. In low-gain conditions (1, 10, and 50 RON), almost all participants returned the banknote, indicating that they were more preoccupied with restoring self-congruence than their personal gain, while in high-gain conditions (100 and 500 RON) only 88% and 80.3% did do (see Table 2). Therefore, we conducted a chi-square test to investigate whether the value of the banknote moderated participants’ subsequent pro-social behavior toward the second confederate. Results indicated that the rate of return was significantly associated with the value of the lost banknote, *χ*^2^(4) = 13.283, *p* = 0.01, *V* = 0.246 (large effect). The rates of return were significantly higher in the 1 RON condition than in the 500 RON condition (*Z* = 2.101, *p* = 0.017); in the 10 RON condition than in the 500 RON condition (*Z* = 2.449, *p* = 0.007); and in the 50 RON condition than in the 100 RON (*Z* = 1.672, *p* = 0.047) and 500 RON (*Z* = 2.523, *p* = 0.005) conditions. Moreover, the rate of return in the 10 RON condition was higher than in the 100 RON condition, though this was just above the threshold of statistical significance (*Z* = 1.615, *p* = 0.052). There were again no significant differences between 1, 10 and 50 RON conditions. Therefore, the pattern of results is similar to the previous one and offers support for our second hypothesis (H2).

**Table 2:** Cross-tabulation of values of the “lost” banknote and pro-social behavior toward the second confederate (*N* = 219).

Pro-social behavior (DV2)	Value of banknote						*χ* ^2^	*df*	*V*
	1 RON	10 RON	50 RON	100 RON	500 RON	Total			
Returned the banknote	29 (96.7%)	37 (97.4%)	39 (97.5%)	44 (88.0%)	49 (80.3%)	198 (90.4%)	13.283^**^	4	0.246
Appropriated the banknote	1 (3.3%)	1 (2.6%)	1 (2.5%)	6 (12.0%)	12 (19.7%)	21 (9.6%)			

*V = effect size (Cramer’s V coefficient) and RON, Romanian currency (Leu)*.

** *p = 0.01*.

Regarding the volume of explanations produced by the participants in relation to the value of the banknote, Table 3 indicates the means and SD for this variable for the participants who returned the banknote, for the ones who did not and for the global sample.

**Table 3:** Means and SD of the number of words in the post-experimental interviews.

Value of banknote	Participants who returned the banknote (*N*_1_ = 368)			Participants who appropriated the banknote (*N*_2_ = 219)			Global sample (*N* = 587)		
	*N*	*M*	*SD*	*N*	*M*	*SD*	*N*	*M*	*SD*
1 RON	87	13.57	12.49	30	21.17	9.58	117	15.51	1.54
10 RON	85	12.40	9.99	38	23.82	15.56	123	15.93	1.51
50 RON	77	13.65	15.55	40	19.85	11.43	117	15.77	1.51
100 RON	68	14.22	12.05	50	24.34	17.26	118	18.51	1.47
500 RON	51	15.80	14.51	61	38.66	26.11	112	28.26	1.51
Total	368	13.75	12.78	219	26.98	19.67	587	18.68	16.99

**Figure 1:**
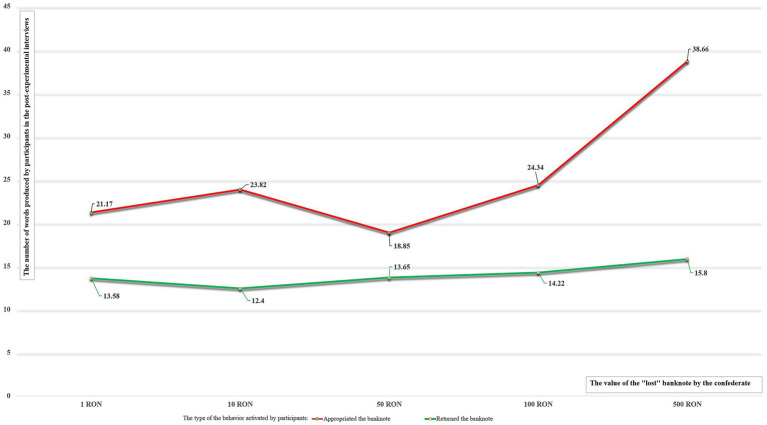
Number of words produced in the post-experimental interviews as a function of returning behavior and value of the lost banknote.

To test our third hypothesis, we conducted an independent samples t-test by which we compared the mean number of words produced by the participants who initially stole the banknote (*M* = 26.980, *SD* = 19.666) with that of the participants who returned it to its rightful owner (*M* = 13.750, *SD* = 12.872). A check of normality was conducted by inspecting skewness (0.952 and 2.110, respectively) and kurtosis (0.896 and 6.643, respectively) values for both groups, which revealed no serious violations, as all values were between the limits of −3 to 3 for skewness and −7 to 7 for kurtosis (see Hair et al., 2010). The difference between groups was significant, *t*(585) = 9.846, *p* < 0.001, *d* = 0.796 (large effect), indicating that self-discrepant participants used significantly more words to explain their behavior than those who were not self-discrepant. Our third hypothesis (H3) was therefore supported by the data. Exploratively, we investigated the impact of value of money on the number of words produced. For this, we used a factorial ANOVA with pro-social behavior toward the first confederate and value of money as predictors. The main effect of pro-social behavior *F*(1) = 76.854, *p* < 0.001, *η_p_*^2^ = 0.118 was significant, indicating that, as in the previous test, participants who stole the money produced significantly more words than those who did not. This difference in pro-social behavior explained 11.8% of the variance in the number of words produced by the participants. The analysis revealed also a main effect for value, *F*(4) = 8.665, *p* < 0.001, *η_p_*^2^ = 0.057, indicating that the number of words produced was dependent on the value of the banknote. *Post-hoc* tests with Tukey correction indicated significant differences between the condition of the most valuable banknote (500 RON) and all other conditions [mean difference (500 RON—100 RON) = 9.74, *t* = 4.513, *p* < 0.001; mean difference (500 RON—50 RON) = 12.48, *t* = 5.769, *p* < 0.001; mean difference (500 RON—10 RON) = 12.32, *t* = 5.765, *p* < 0.001; mean difference (500 RON—1 RON) = 12.73, *t* = 5.885, *p* < 0.001], while there were no significant differences between the other conditions. The value of the banknote explained 5.7% of the variance in the number of words. There was also a significant interaction between pro-social behavior toward the confederate and the value of the banknote, *F*(4) = 5.109, *p* < 0.001, *η_p_*^2^ = 0.034, indicating that the increase in the volume of explanations with the value of the banknote depends on the type of behavior participants engaged in (returned vs. stole the banknote). There was no change in the number of words for different values of the banknote for those who returned the money (*F*(4) = 0.584, *p* = 0.675), while for self-discrepant participants the number of words increased with the value of the banknote, *F*(4) = 8.958, *p* < 0.001.There were significant differences in the number of words between the 500 RON condition and all the other conditions [mean difference (500 RON—100 RON) = 14.32, *t* = 4.085, *p* = 0.001; mean difference (500 RON—50 RON) = 18.806, *t* = 5.032, *p* < 0.001; mean difference (500 RON—10 RON) = 14.84, *t* = 3.909, *p* = 0.001; mean difference (500 RON—1 RON) = 17.49, *t* = 4.27, *p* < 0.001] and no significant differences between the other conditions (see Figure 1).

Regarding the results obtained from the thematic analysis, our study does not claim to be representative; it is more concerned with the in-depth process of meaning creation by ordinary people in real-life interactions. The referential themes were grouped in a portfolio of seven categories (see Table 4 for the English version, and Supplementary Material, for the original version, in Romanian). The narrative that appeared with the highest incidence was *non-informative messages* (*N* = 157 from a total of 587 interaction), which covered routinely expressed messages, like a salute or a brief refuse of the dialogue, regularly formulated in a few words. Therefore, more than a quarter of participants decided not to communicate any significant informative message in their final interaction with the second confederate. The next most mentioned themes were *interpersonal and functional cynicism* (*N* = 101), *explicitly/ostentatiously normative* (*N* = 92), *mercy and support* (*N* = 73), *implicitly normative* (*N* = 71), *absurd/incoherent explanations* (*N* = 59), while *recognizing/assuming the mistake* (*N* = 38) was the least mentioned theme.

**Table 4:** IPA matrix of referential themes and participants’ statements (global sample, *N* = 587).

Theme	*N* (%)	Relevant examples
1. Non-informative messages	157 (26.74%)	‒ “I’m in a hurry, goodbye”/“That’s it”/“I’m sorry, I am late”/“Goodbye, I’m in a hurry, sorry”/“Hello”/“I cannot”/“Yes”/“No”/“Give me a break”/“I cannot now”/“Good day” etc.
2. Interpersonal functional cynicism	101 (17.21%)	‒ “And, what’s the problem?”/“Obviously I took the money, because I also get cheated in life, not rewarded. Was I supposed to be the loser when for once I have the occasion to be the winner?”/“After all, most would have done as I did!”/” I do not earn 500 lei in half a month, so what was I supposed to do?!”/“I do not give a damn about your research, the only things that matters is to win here and now! Do you think someone is doing charity to me?”/“If you receive such mana from heaven you must be a loser to blow it away!”/“Look, I did something that others would have done to me, so I do not see the problem?!”/“I bended the rules gracefully, because I just wasn’t going to leave it to another hunger-bitten to take it. Am I the one to feed a hunger-bitten?”/“In short, if everyone steals from me, I am not going to play generous!”/“The thief goes hand in hand with the lord”/“Giving others a bum deal earns somebody a living” etc.
3. Explicitly/Ostentatiously normative	92 (15.67%)	‒ “To be honest is a golden rule in life”/“I’ve always done the right thing and I want to go to sleep at peace every night”/“I’ve never stolen in my life”/“Well, if we all stole from each other, what would be left of this country?… Not that there’s much left…”/“That’s what we all should do! I hope that’s also what happened!”/“Honesty is something that should never be given away, for nothing!”/“We must always help each other, because that’s what my parents taught me. Otherwise, it will be very bad for all of us”/“Mister, whoever steals others steals himself!” etc.
4. Mercy and support	73 (12.43%)	‒ “I wonder how others could have stolen from a penniless?”/“A poor old man… he should have been helped”/“Look at him, he’s close to dropping dead. If he saw that he was really left without 100 lei, he would have died on the spot. How was I supposed to seal from such a guy?”/“Well, look how needy he is!”/“How was I supposed not to give him his money back when you clearly see he needs it?”/“That poor old guy… I’d lose my right arm if I’d steal from this guy. It was a must to help him” etc.
5. Implicitly normative	71 (12.09%)	‒ “This is what you should do”/“I could not have done it otherwise”/“It’s natural”/“But what would you suggest me to do?”/“To be such a jerk to steal a poor old man, is hard to imagine”/“Well, that’s the order of things, to give back what is not yours” etc.
6. Absurd/Incoherent explanations	59 (10.05%)	‒ “I knew it was a worthless piece of paper”/“I knew that if he looked at me and looked after his money, I would have returned it to him”/“I did not realize it”/“I thought he was a dirty peasant, what do you want from such a guy?”/“I went ahead, I just wasn’t going to go back …” etc.
7. Recognition and assuming the mistake	38 (6.47%)	‒ “I’m truly sorry”/“I really do not know what happened to me”/“I’m sorry”/“Sorry, that’s it”/“I was a lame brain, but I’m sorry. Look, mister, your money back (n. ns.—it is pointed out that it is not “real” money)… Uff, I’m sorry mister…”

We performed a chi-square test to investigate whether pro-social behavior toward the first confederate influenced participants’ propensity for specific themes in their narratives. Test results evidenced significant differences in selecting specific themes, depending on participants’ behavior toward the first confederate: *χ*^2^(6) = 451.276, *p* < 0.001, generating a very large effect, *V* = 0.877 (Cohen, 1988, p. 222; see Table 5). While those who returned the banknote had narratives mostly dominated by normative considerations (44.3%), non-informative messages (33.4%) and mercy and support for the victims (19.8%), those who decided to steal the banknote evoked cynicism (46.1%) or offered absurd or incoherent explanations for their behavior (22.8%) and only in 17.4% of the cases they recognized their mistake.

**Table 5:** Cross-tabulation of pro-social behavior toward the first confederate and the themes generated during the post-experimental interview (*N* = 587).

Themes	Pro-social behavior toward the first confederate					
	Returned the banknote	Appropriated the banknote	Total	*χ* ^2^	*df*	*V*
Non-informative messages	123 (33.4%)	30 (13.7%)	153 (26.1%)	451.276^***^	6	0.877
Implicitly normative	71 (19.3%)	0 (0%)	71 (12.1%)			
Explicitly/Ostentatiously normative	92 (25.0%)	0 (0%)	92 (15.7%)			
Functional interpersonal cynicism	0 (0%)	101 (46.1%)	101 (17.2%)			
Absurd/Incoherent explanations	9 (2.4%)	50 (22.8%)	59 (10.1%)			
Mercy and support	73 (19.8%)	0 (0%)	73 (12.4%)			
Recognizing/Assuming the mistake	0 (0%)	38 (17.4%)	38 (6.5%)			
Total	368	219	587			

*V = effect size (Cramer’s V coefficient)*.

*** *p < 0.001*.

Similarly, to test whether participants’ subsequent pro-social behavior toward the second confederate influenced their tendency to produce specific themes, we performed a chi-square test only on the sample of participant who initially stole the banknote. Our outcomes indicated that there were significant differences in selecting specific themes, depending on the activated behavior in relation to the second confederate: *χ*^2^(3) = 91.581, *p* < 0.001. The effect, *V* = 0.647, was again a very large one (see Table 6). Thus, more than 2/3 of the participants who stole the banknote (68.9%) produced in the interaction with the second confederate either a cynical or an absurd/incoherent explanation regarding their previous behavior. In the same time, only less than 1/5 of participants (17.4%) from this category decided to assume the mistake and express remorse in their spontaneous narrative provided to the second confederate.

**Table 6:** Cross-tabulation of pro-social behavior toward the second confederate and the themes generated during the post-experimental interview (*N* = 219).

Themes	Pro-social behavior toward the second confederate					
	Returned the banknote	Refused to return the banknote	Total	*χ* ^2^	*df*	*V*
Non-informative messages	13 (6.6%)	17 (81%)	30 (13.7%)	91.581^***^	3	0.647
Implicitly normative	0 (0%)	0 (0%)	0 (0%)			
Explicitly/Ostentatiously normative	0 (0%)	0 (0%)	0 (0%)			
Functional interpersonal cynicism	101 (51%)	0 (0%)	101 (46.1%)			
Absurd/Incoherent explanations	46 (23.2%)	4 (19%)	50 (22.8)			
Mercy and support	0 (0%)	0 (0%)	0 (0%)			
Recognizing/Assuming the mistake	38 (19.2%)	0 (0%)	38 (17.4%)			
Total	198	21	219			

*V = effect size (Cramer’s V coefficient)*.

*** *p < 0.001*.

## Discussion

The present study investigated pedestrians’ pro-social behavior toward an unknown person, who supposedly lost a banknote of different values, through a field experiment organized in a public space. Our study used both a quantitative approach, through which we measured participants’ pro-social behavior toward the confederate who lost the banknote, their subsequent pro-social behavior toward a second confederate who exposed their immoral behavior (for those that initially stole the banknote) and the number of words they produced in their explanations, as well as a qualitative one, through which we explored the themes emerging from their interviews and their relation with participants’ previous behavior. Based on SEUT, we expected to see a progressive reduction in participants’ pro-social behavior toward the confederate who lost the money and in their subsequent behavior toward the second confederate, as their the potential economic gain increased, while based on self-discrepancy and face-negotiation theory we expected to see a higher volume of explanations for those participants who initially appropriated the money than for those who returned it to its rightful owner.

Firstly, regarding global pro-social behavior, 63% of the total number of participants in our sample acted pro-socially and returned the banknote to the person who lost it. This proportion is remarkably similar to the one in Cohn et al. (2019), who found in a sample of 400 Romanians from seven cities (including Timisoara), that the rate of returning “lost” wallets was 63% in the money condition (when they contained 28 RON) and 50% in the no money condition. Compared to Cohn et al. (2019) study, however, in which the amount of money was fixed, the value of the lost money in our study was manipulated, to test its impact on returning rates. Consistent with SEUT, our results show that when the economic gain was experimentally increased, participants’ propensity to act in a pro-social manner decreased significantly; their behavior was the least pro-social in the maximum gain condition (500 RON) and the most pro-social in the minimum gain condition (1 RON). Therefore, the majority of participants (almost 75%) behaved pro-socially toward an unknown person needing help in a public space when there was almost no economic gain, yet this percentage dropped to less than half (45%) when their own immediate gain became substantial (approximately 100 euro). Even though no significant differences were detected between the 1, 10, and 50 RON conditions, possibly due to a lack of statistical power, the proportion of those who were willing to help another person in need decreased progressively with the increase in the value of the lost money. These results reconfirm the findings of previous studies (e.g., Newman, 1979; Armantier and Boly, 2011; Castillo et al., 2014), which found that increased benefits lead to less pro-social behavior. However, other studies in the literature (e.g., Azar et al., 2013; Cohn et al., 2019) found that greater rewards yielded more pro-social behavior, which might be partly explained by the non-anonymous nature of participants in these studies. While in the present study participants’ anonymity was guaranteed by the place of interaction (a busy public space) and by the fact that no interaction with participants took place before their behavior was measured, in Cohn et al. (2019) study participants were entrusted lost wallets in a highly personal setting (i.e., at their workplace), while in Azar et al. (2013) study they were (in some cases even regular) customers of a restaurant who had previously established some rapport with the waiter on whom they were offered the opportunity to cheat.

Regarding participants’ subsequent pro-social behavior toward the second confederate who politely asked them to return the banknote, results show that activating self-discrepancies motivated almost all of those who initially appropriated the banknote to return it in the 1 RON (96.7%), 10 RON (97.4%) and 50 RON (97.5%) conditions, while 88% and 80.3% returned it in the 100 RON and 500 RON conditions, respectively. Such results indicate that, when self-discrepancies were activated, the majority of participants significantly improved their behavior, a result that contradicts the moral consistency evidenced in some studies (e.g., Martens et al., 2010). Thus, becoming aware of discrepancies from personal or societal standards caused most of the tempted individuals to behave inconsistently and revert their previous anti-social behavior. This helped them to restore their sense of morality in two different ways: participants engaging in self-deceiving strategies after their initial moral transgression (i.e., those avoiding the recognition of the discrepancy between their initial behavior and their moral standards, Batson et al., 1997; see also Rustichini and Villeval, 2014) restored moral congruence by acting in line with their own values, whereas the reverting behavior of those participants using other-deceiving strategies can be understood as a form of social signaling and a desire to appear moral in the eyes of others rather than an authentic desire to be moral. This moral hypocrisy (Batson et al., 1997, 1999) through which people are concerned with appearing moral while also benefiting from dishonesty, was also evidenced in the narratives of the participants who acted dishonestly, which indicated that almost 83% of them did not recognize their immoral behavior publicly but tried to justify it instead. Overall, the morally inconsistent behavior evidenced in the present study is in line with other studies that identified a propensity to engage in compensatory behaviors as a response to previous moral transgressions (e.g., Jordan et al., 2011; Mulder and Aquino, 2013). However, our results show that this process does not happen equally for everybody, but it is moderated by personal benefit. While almost all participants reverted their dishonest behavior and returned the previously appropriated banknote in the 1, 10, and 50 RON conditions, almost 20% refused to do so in the 500 RON condition, when their personal gain was significant. When economic gain becomes subjectively significant, people’s desire to profit from dishonest behavior increases, yet so does the threat to one’s self-image (Cohn et al., 2019). Therefore, it seems that in such high-gain circumstances, people may be more willing to incur the discomfort of self-discrepancy and the cost to their self or public image in exchange for economic benefit, while in low-gain situations their main motivation is to restore moral congruence by engaging in compensatory moral behavior. This malleability in moral behavior due to situational influences attests to the opportunistic, self-serving use of morality, through which individuals balance moral considerations with their self-interested motivations (see also Rustichini and Villeval, 2014).

Making participants aware of their moral transgression (i.e., activating self-discrepancies) not only improved their subsequent behavior, but also motivated them to engage in a cluster of communicative behaviors to cover up their immoral behavior and negotiate their own self-image. Those who initially stole the banknote used on average two times more words to explain their behavior than those who returned it, and this difference was more pronounced for higher values of the banknote. This may indicate that participants’ image in the 500 RON condition was the most threatened by their self-discrepant behavior, which motivated them to engage in face-restoring strategies more than in other conditions. In collectivistic cultures, such as the Romanian one (Hofstede et al., 2010; Gavreliuc, 2011), where an interdependent self-construal pattern is prevalent (Gavreliuc and Ciobotă, 2013; Moza et al., 2021), individuals tend to use more avoidance strategies, less aggressive conflict styles, more obliging and compromising strategies and show more mutual face concern compared to individualistic cultures (Ting-Toomey, 2010). However, the results of our qualitative analysis are in many respects in contradiction with these expectations because, even though they eventually returned the money, the majority to those who initially stole the banknote adopted a cynical (46.1% of them) or absurd or incoherent description of their previous behavior (22.8%) and only in a relatively reduced number of cases they evoked something that could suggest remorse by recognizing and assuming their mistake (in 17.4% of cases), evidencing therefore a desire to appear moral even though they failed to admit their dishonest behavior. Thus, many of them refused to adopt an obliging or compromising strategy and were guided more by an egoistic face concern, without much consideration for the other. Most of the narratives produced by these participants stressed their interpretation in terms of moral hypocrisy, spontaneously activated in few memorable statements (e.g., “Obviously I took the money, because I also get cheated in life, not rewarded. Was I supposed to be the loser when for once I have the occasion to be the winner?,” or “I do not give a damn about your research, the only things that matter is to win here and now! Do you think someone is doing charity to me?,” or “The thief goes hand in hand with the lord”). Turning to the issue of cross-cultural consistency, participants who behaved pro-socially by returning the money to the person who lost it had narratives that were more dominated by morality (mercy and support) or were explicitly or implicitly normative.

Pro-social behavior was shown to vary considerably across different cultures. For instance, in Cohn et al. (2019) study, the incidence of returning the lost wallets in the money condition was the highest in countries like Sweden (82%), Denmark (82%), Norway (80%) or Switzerland (80%), while in Mexico and Peru it was only 16% and 14%, respectively. The relatively low rate of pro-social behavior in our study could be explained by the persistence of a social background characterized by a high level of social cynicism (Dincă and Iliescu, 2008; Gavreliuc and Gavreliuc, 2018), generalized interpersonal and institutional distrust (Gavreliuc, 2011; Friedlmeier and Gavreliuc, 2013; Voicu, 2020), a prevalent pattern of negative interactional experiences with others (Mihăilescu, 2017; Gavreliuc et al., 2021) and the prevalence of traditionalist and conservative values (Voicu and Voicu, 2007; Gavreliuc, 2011), associated with a visible decline of solidarity toward the “(ordinary) people from Romania” (Rusu, 2020, p. 66). Therefore, this egoistic concern could be interpreted as a functional way of thinking and acting (Gavreliuc et al., 2009) in a society characterized by mistrust and low normative climate, by routinely activating a mechanism of tolerated deviance (Stebbins, 2012). In a social context characterized by these features, the propensity to act pro-socially in spontaneous interpersonal interactions with strangers can prove to be too costly for a lot of individuals.

## Limitations and Future Studies

The present study has a few limitations worth mentioning. A first possible methodological limitation lies in the way facework was operationalized. We considered the higher number of words expressed by the participants that stole the banknote as a sign of their engagement in facework, yet there is a possibility that this represents a proxy for other type of behavior. One way to approach this dilemma is by inspecting the themes emerging from participants’ interviews, which show that at least three of the four evoked themes (i.e., interpersonal functional cynicism, absurd explanations, and recognizing/assuming the mistake) could be related to facework. Participants adopting a cynical attitude generally gave a “lesson about life’s unfairness” to the confederate interviewing them, possibly as a way of emphasizing their “normal” behavior, while the behavior of those using more words to offer absurd explanations could be understood as a symbolic act to exculpate oneself.

A second limitation that could have impacted the results of the present study is related to the fake banknotes used. Even though they were almost identical to the real ones and differences could not be identified at first sight (as revealed also in the pilot study), there is the possibility that some participants identified them as fake money, which could have affected their behavior. However, it is likely that this realization (if it happened) actually increased the global rate of pro-social behavior, as participants could tell whether the banknotes were real or not only after they picked them up from the ground. In this case, participants had no reason to retain the money and most probably returned them to their rightful owner. It is also worth adding that participants had a chance to justify (to the second confederate) keeping the note on the grounds that it was worthless, but it appears that they did not.

Another limitation resides in the fact that we assumed that confronting participants with their own immoral behavior will generate self-discrepant states and their associated discomfort, yet we did not measure self-discrepancies or participants’ emotional states. Also, the theoretical support used to understand and interpret the findings (i.e., self-discrepancy theory) is just one of the possible theoretical lenses through which such findings can be viewed. The behavior of participants can also be understood as form of cognitive dissonance (Festinger, 1957), incongruity (Osgood and Tannenbaum, 1955), or other forms of inconsistent behavior.

Because pro-social behavior is also determined by cultural factors and can vary considerably across different cultures (see Cohn et al., 2019), it is not yet clear whether the same moderating effect of economic gain on subsequent pro-social behavior can be observed in cultures with a high vs. a low pro-social orientation. In order to understand whether the impact of economic interest in anonymity conditions on pro-social behavior is universally manifested and to the same degree, more research on cross-cultural samples is needed.

Finally, it is clear that pro-social behavior is a complex phenomenon that cannot be explained solely by self-interest motivations (Gibson et al., 2013; Abeler et al., 2014). Future studies should attempt to manipulate further the interplay between costs and benefits by varying different situational (e.g., anonymity and presence of peers) and individual factors (e.g., the salience of moral identity or religiosity) and investigate their interaction with economic interest, to be able to delineate the boundary conditions of such influences on pro-social behavior. A more complex design could also vary the identity of the confederate and its associated stereotypes (e.g., a business person and an exponent of a sexual or religious minority), the type of residence (rural vs. urban), the nature of the place of interaction (private vs. public one), or the type of task required (volunteer vs. non-volunteer one), in order to extend and deepen the analysis. At the same time, we have to caution about generalizing the present results to all types of pro-social behavior. As the task in our study involved low engagement, it is unclear whether in circumstances of higher personal involvement the same effect of economic benefit on behavior can be observed. Future studies will have to investigate this possibility.

## Conclusion

The present field experiment identified a moderating effect of economic interest on pro-social behavior toward a stranger losing money on the street. Thus, when their potential gain was larger, participants were less likely to return a lost banknote to its rightful owner than when their potential gain was smaller.

Activating a self-discrepant state in those who initially appropriated the banknote, by recognizing their immoral behavior, led them to improve their subsequent behavior and return the stolen banknote in most cases.

However, this effect was again moderated by economic interest such that participants were less likely to return the money in high-gain than in low-gain situations. Moreover, to cover their behavior and restore their threatened image, those who initially stole the money were more likely to engage in a face-negotiation process, during which they used significantly more words to explain their behavior compared to those individuals who acted pro-socially and returned the money. A qualitative analysis of their interviews also revealed completely different themes in their narratives than in the narratives of those who decided to return the money. The present study provides new evidence on the moderating effect of financial interest on pro-social behavior, in a context of public anonymity, with ostensibly high financial stakes involved and in an under-studied culture.

## Data Availability Statement

The datasets presented in this study can be found in online repositories. The names of the repository/repositories and accession number(s) can be found at: OSF (Open Science Foundation): https://doi.org/10.17605/OSF.IO/H2U5W.

## Ethics Statement

The studies involving human participants were reviewed and approved by Scientific Committee of the Center for Social Diagnosis from the Faculty of Sociology and Psychology from the West University of Timisoara. Written informed consent for participation was not required for this study in accordance with the national legislation and the institutional requirements.

## Author Contributions

AG and DG contributed to the conception and design of the study, coordinated the field experiment, organized the database, and wrote the first draft of the manuscript. AG, DG, and AS performed the statistical analysis. All authors contributed to the article and approved the submitted version.

## Conflict of Interest

The authors declare that the research was conducted in the absence of any commercial or financial relationships that could be construed as a potential conflict of interest.

## Publisher’s Note

All claims expressed in this article are solely those of the authors and do not necessarily represent those of their affiliated organizations, or those of the publisher, the editors and the reviewers. Any product that may be evaluated in this article, or claim that may be made by its manufacturer, is not guaranteed or endorsed by the publisher.
