# Supplementary material for: To Steal or Not to Steal: Self-Discrepancies as a Way to Promote Pro-social Behavior: The Moderating Role of Self-Interest
Source: Front Psychol. 2022 Apr 27;13:748298. doi: 10.3389/fpsyg.2022.748298 (PMC9092975; doi:10.3389/fpsyg.2022.748298)
Supplement: Supplementary file 1 [file Data_Sheet_1.PDF]

Supplemental material

Thematic references in Romanian

Matricea IPA care prezintă temele generate de către subiecți în cadrul interviului post-experimental (eșantionul global, N = 587)

Teme referențiale	N (%)	Formule discursive (exemple semnificative)
(1) Mesaje non-informative	157 (26.74%)	-“La revedere, mă grăbesc” / ”asta e” / ”îmi pare rău, întîrzii undeva” / ”servus, sunt grăbit, sorry” / ”salut” / ”nu pot” / ”da” / ”nu” / ”mai lasă-mă” / ”nu pot acum” / ”bună ziua” șa.
(2) Cinism interpersonal funcțional	101 (17.21%)	- ”Și care e problema?” / ”Normal că am luat banii, căci mie de regulă mi se ia în viață, nu mi se dă. Ce, era să fiu fraier cînd mi s-a oferit ocazia să fiu și eu o dată cîștigătorul?” / ”La urma urmei, majoritatea ar fi făcut ca mine!”/ ”Eu nu cîștig 500 de lei în juma’ de lună, așa că ce era să fac?!” / ”Mi se rupe de cercetarea voastră, singurul lucru care contează e să mă *scot* acum și aici! Ce, cineva face acte de binefacere cu mine?” / ”Dacă se ivește o *pleașcă*, ești fraier dacă îi dai cu piciorul!”/ ”Uite ce e, am făcut ceva ce și alții mi-ar fi făcut mie, așa că nu văd problema?!” / ”Am *cotit-o* și eu cu grație, că doar

		nu era să las la alt *fomist* s-o *șparlească.* Ce, hrănesc eu fomiștii?”/ ”Pe scurt, mi se pare că dacă toți mă fură, doar nu o să fac eu pe generosul!” / ”Hoția merge în mînă cu domnia” / ”*Țapa’* scoate omul!” șa.
(3) Normativitate explicită / ostentativă	92 (15.67%)	- ”A fi cinstit e regula de aur în viață” / ”Dintotdeauna am făcut ceea ce trebuie și vreau să mă culc liniștit în fiecare seară” / ”Eu nu am furat niciodată în viață” / ”Păi dacă toți ne-am fura pe față, ce ar mai rămâne din țara asta?... Nu că a rămas tare mult....” / ”Asta trebuia să facem toți! Sper că așa s-a și întîmplat!” / ”Cinstea e ceva ce nu se vinde niciodată, pentru nimic!” / ”Trebuie să ne ajutăm unii pe alții mereu, că pe mine așa m-au învățat ai mei. Altfel, va fi *vai și-amar* de capul nostru” / ”Nene, cine fură pe alții se fură pe sine! șa.
(4) Milă / îndurare & ajutor	73 (12.43%)	- “Stau să mă întreb cum au putut alții să fure un nenorocit?” / ”Un amărît de bătrîn, ..., trebuia ajutat” / ”Uită-te la el, mai puțin și *dă colțul.* Dacă mai și vedea că a rămas cu adevărat fără 100 de lei, crăpa *live.* Cum să-l furi?” / ”Păi uite ce năcăjit este!” / ”Cum să nu-i dai banii înapoi, cînd vezi clar că are nevoie de ei? / ”Vai de mama lui de moș, păi îmi cădea mîna dacă i-o *făceam*, era musai să-l ajut” șa.

(5) Normativitate implicită (rînduiala)	71 (12.09%)	- ”Păi, așa se cade” / ”Nu puteam altfel” / ”E firesc” / ”Dar cum ați fi vrut să fac?” / ”Chiar așa nesimțit să furi un bătrîn nenorocit e greu de imaginat” / ”Păi așa e rînduiala, să dai înapoi ceea ce nu e al tău” șa.
(6) Explicații absurde / incoerente	59 (10.05%)	- ”Știam că e o hîrtie fără valoare” / ”Știam că mă dacă mă va privi și se va uita după banii lui, i-aș fi înapoiat” / ”Nu mi-am dat seama” / ”Credeam că e un țărănist murdar, ce vrei de la el?” / ”Am mers înainte, că doar nu era să merg înapoi…” șa
(7) Recunoașterea / asumarea greșelii	38 (6.47%)	- ”Îmi pare sincer rău” / ”Chiar nu știu ce mi-a venit” / ”Regret” / ”Scuze, asta e” / ”Atît m-a dus capul, dar îmi pare rău. Uite, nene, banii tăi (n. ns. – i se atrage atenția că nu sunt bani ”adevărați”)… Uff, iartă-mă nene…”
