# Supplementary figures and images for: To Steal or Not to Steal: Self-Discrepancies as a Way to Promote Pro-social Behavior: The Moderating Role of Self-Interest
Source: Front Psychol. 2022 Apr 27;13:748298. doi: 10.3389/fpsyg.2022.748298 (PMC9092975; doi:10.3389/fpsyg.2022.748298)

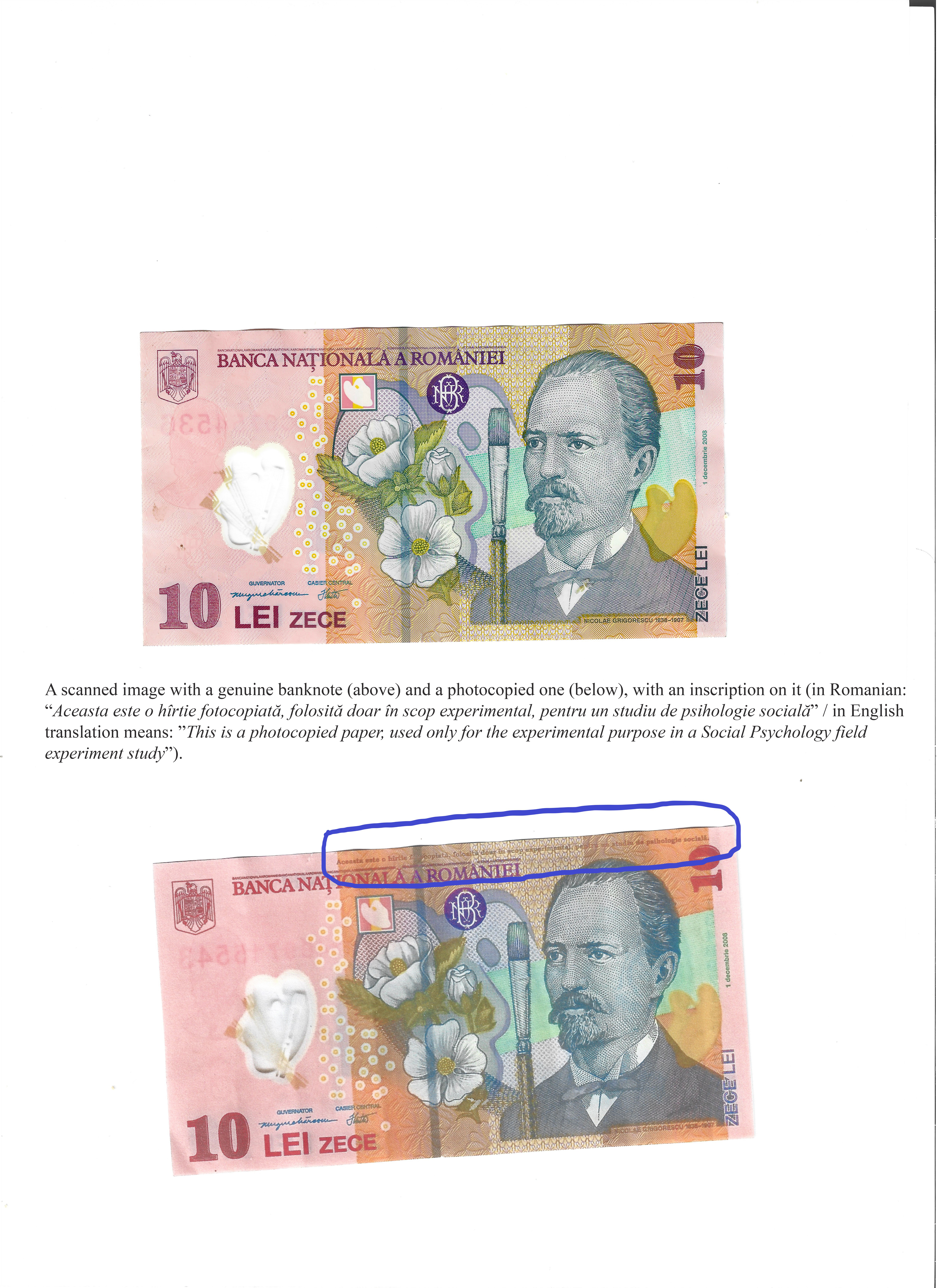

Supplement: Supplementary file 2 [file Image_1.JPEG]
